# Endogenous expression of ASLV viral proteins in specific pathogen free chicken embryos: relevance for the developmental biology research field

**DOI:** 10.1186/1471-213X-10-106

**Published:** 2010-10-18

**Authors:** Minda M McNally, Karl J Wahlin, M Valeria Canto-Soler

**Affiliations:** 1 Wilmer Eye Institute, Department of Ophthalmology, Johns Hopkins University School of Medicine, 400 N Broadway, Baltimore, MD USA

## Abstract

**Background:**

The use of Specific Pathogen Free (SPF) eggs in combination with RCAS retrovirus, a member of the Avian Sarcoma-Leukosis Virus (ASLV) family, is of standard practice to study gene function and development. SPF eggs are certified free of infection by specific pathogen viruses of either exogenous or endogenous origin, including those belonging to the ASLV family. Based on this, SPF embryos are considered to be free of ASLV viral protein expression, and consequently in developmental research studies RCAS infected cells are routinely identified by immunohistochemistry against the ASLV viral proteins p19 and p27. Contrary to this generally accepted notion, observations in our laboratory suggested that certified SPF chicken embryos may endogenously express ASLV viral proteins p19 and p27. Since these observations may have significant implications for the developmental research field we further investigated this possibility.

**Results:**

We demonstrate that certified SPF chicken embryos have transcriptionally active endogenous ASLV loci (*ev loci*) capable of expressing ASLV viral proteins, such as p19 and p27, even when those *loci *are not capable of producing viral particles. We also show that the extent of viral protein expression in embryonic tissues varies not only among flocks but also between embryos of the same flock. In addition, our genetic screening revealed significant heterogeneity in *ev loci *composition even among embryos of the same flock.

**Conclusions:**

These observations have critical implications for the developmental biology research field, since they strongly suggest that the current standard methodology used in experimental studies using the chick embryo and RCAS vectors may lead to inaccurate interpretation of results. Retrospectively, our observations suggest that studies in which infected cells have been identified simply by pan-ASLV viral protein expression may need to be considered with caution. For future studies, they point to a need for careful selection and screening of the chick SPF lines to be used in combination with RCAS constructs, as well as the methodology utilized for qualitative analysis of experimental results. A series of practical guidelines to ensure research quality animals and accuracy of the interpretation of results is recommended and discussed.

## Background

The chicken embryo has historically been the animal model *par excellence *to study vertebrate development, and its potential was significantly enhanced with the generation of the RCAS retrovirus vectors [1,2]. Among the main characteristics that make these vectors so useful for developmental studies are: i) ability to efficiently infect proliferating cells; ii) integration in the genome of infected cells; and iii) ability to efficiently replicate [3]. As a result, RCAS vectors offer a powerful system ensuring long-term expression of the gene of interest and its transmission to daughter cells, coupled to the production of new replication competent viral particles that rapidly spread infection to proliferating neighbor cells.

The RCAS vectors (Replication Competent ALV LTR with a Splice acceptor) are laboratory-derived vectors engineered from the Rous Sarcoma Virus (RSV), a naturally-derived retrovirus originated from the Avian Leukosis Virus (ALV) [3]. Members of the Avian Sarcoma-Leukosis Virus (ASLV) family, which include the ALVs, RSVs and RCAS viruses, are classified into 6 subgroups (A-E and J) based on antigenic characteristics of their envelope (*env*) glycoproteins. They are also classified as being either exogenous or endogenous; thus ASLV subgroups A-D and J are "exogenous": naturally occurring viruses that can be transmitted either vertically from dam to progeny through the egg, or horizontally from bird to bird, while members of the ASLV subgroup E are "endogenous" viruses: copies of exogenous retroviruses that have been integrated into the host germ line cells and are transmitted genetically in a Mendelian fashion [4]. All members of the ASLV family share highly similar sequences for all the viral proteins except for the region encoding the subgroup specific gp85 surface envelope glycoprotein, which determines the ASLV subgroup classification and subgroup infection-specificity [4].

Endogenous proviruses of the ASLV family are encoded by the so-called endogenous viral (*ev) loci *[5]. Most chicken strains contain *ev loci*, and in the particular case of White Leghorn chickens, the strain most commonly used in developmental studies, more than 20 different *ev loci *(*ev-1 through ev-23*) have been already identified [6-8]. Most of these *ev loci *are structurally incomplete and therefore do not encode all sequences necessary for production of infectious ASLVE viral particles [6-8]. White Leghorn chickens free of *ev loci *are extremely rare. As reported by Zhang and colleagues [9], there are only three known chicken lines free of *ev loci*: the East Lansing Line 0 and Line 0.TVB*S1, both from the US Department of Agriculture, and the Canadian line WG.

Developmental biologists utilizing the chicken model system routinely use eggs designated as "Specific Pathogen Free" (SPF), which are derived from controlled breeding flocks certified free of infection by specific pathogen viruses, including those belonging to the ASLV family. In such studies, the use of SPF embryos is required for at least three main reasons: i) in an infected cell, expression of the envelope glycoprotein blocks the receptors on the surface of the cell, inducing the so called 'receptor interference' response. Thus, *pre-infection by exogenous ASLV viruses will preclude subsequent infection with experimental RCAS viruses of the same subgroup *[3]; ii) retroviruses readily recombine with closely related retroviruses of either exogenous or endogenous origin, thus raising the possibility of *undesirable recombination events between the RCAS vector and ASLV viruses *[3]; and iii) experimentally RCAS-infected cells are routinely identified by expression of viral proteins such as p19 or p27, which are also expressed by all members of the ASLV family [4]. Therefore, *pre-infection with ASLV virus would interfere with proper identification of RCAS infected cells and/or tissues*. Therefore, the fact that most available chicken lines, including SPF lines, have *ev loci *in their genome that could produce endogenous viral proteins in addition to the ectopically expressed RCAS-derived proteins, is of direct relevance to this line of work.

During a series of experiments designed to overexpress different genes of interest by RCAS viral transduction, we observed what appeared to be endogenous expression for the viral proteins p19 and p27 in untreated SPF embryos. Here we demonstrate that certified SPF chicken embryos have transcriptionally active *ev loci *capable of expressing ASLV viral proteins such as p19 and p27 to levels detectable by immunohistochemical analysis even when those *loci *are not capable of producing complete infectious viral particles. We also show that the extent of viral protein expression in embryonic tissues varies not only among flocks but also between embryos of the same flock. In addition, our genetic screening revealed significant heterogeneity in *ev loci *composition even among embryos of the same flock. The main purpose of this report is to raise awareness among developmental biology researchers, about the need for careful selection and screening of the chicken SPF lines to be used in combination with RCAS constructs, as well as the methodology utilized for qualitative analysis of experimental results. In addition, we recommend some practical guidelines that may help researchers ensure quality of the animals used and accuracy of the interpretation of their results.

## Methods

### Animals

All procedures were performed in accordance with the animal protocols approved by the Animal Care and Use Committee at the Johns Hopkins University. Fertilized White Leghorn SPF certified eggs were obtained from three different commercial breeders and are arbitrarily referred herein as flocks 1 to 4: Flocks 1 and 4, Sunrise Farms, Catskill, NY; Flock 2, Hy-Vac, Adel, Iowa; and Flock 3, Charles River, North Franklin, Connecticut. Embryos were incubated in the laboratory at 37°C and 60% humidity in forced air incubator with periodic rocking. Embryos were always manipulated under conditions ensuring no possible contamination from experimental RCAS viruses or other natural exogenous viruses: incubator and working areas were kept free of possible viral contamination by cleaning with 25% bleach; gloves were always worn when manipulating the eggs; and RCAS-injected eggs and non-injected eggs were kept in different incubators.

### RCAS production and embryo infection

Nuclear localized green fluorescent protein (nucGFP) was created by appending a 3x nuclear localization signal to the GFP C-terminus. nucGFP was then cloned into the pENTR-D-TOPO entry vector, and transferred into RCAS-BP-Y-DV (AddGene; Cambridge, MA) using LR clonase. Endotoxin-free plasmids were prepared using the Qiagen endo-free maxiprep kit. DF-1 cells (ATCC, American Type Culture Collection, Manassas, VA) were grown in high glucose DMEM containing 10% FCS. To prepare virus stocks, dissociated DF-1 cells from one 60 mm dish were resuspended in 3 ml of DMEM and 1 ml of transfection mix (8 μg of RCAS plasmid DNA and 20 μl of Lipofectamine 2000 in DMEM). Cells were subsequently incubated in suspension for 45 min at room temperature and seeded at high density (80-90% confluency) in 60 mm dishes containing DMEM 10%FCS, washed with fresh medium after 3-4 hours and split the following day into 2× 100 mm dishes. Cells were thereafter maintained in medium containing only 1% FCS. Supernatants were harvested after 2 additional days and concentrated RCAS stocks obtained by centrifugation in Amicon Ultra-15 100 kDa Filters (Millipore, Billerica, MA) at 3,000 g at 4°C for 30 minutes. RCAS-nucGFP virus stock (0.5-1 μl) was injected in the neural tube of embryonic day (ED) 2.5 or the amniotic chamber of ED6 chicken embryos. Microinjection was performed as described in [10] with minor modifications. After injection, embryos were allowed to develop until ED8, and the amniotic fluid was harvested as described below. Viral infection was confirmed at the time of harvesting using a fluorescent microscope (Axioplan2; Zeiss, Thornwood, NY) to visualize GFP fluorescence in cells of the amniotic membrane.

### Immunohistochemistry

Embryos were decapitated and the heads processed for immunohistochemical analysis as described [10]. Briefly, whole heads were fixed in 4% paraformaldehyde in PBS for 2-3 hours at room temperature, cryoprotected in a serial sucrose gradient (7, 15 and 25% sucrose in 0.1 M phosphate buffer) and frozen in a mixture of 25% sucrose and OCT (2:1; Ted Pella, Redding, CA). Immunohistochemistry (IHC) was carried out in 10 μm sections. Serial sections, collected every fifth section and spanning the region of the head containing the eyes, were incubated overnight at 4°C in the presence of a first antibody and developed for immunofluorescent detection the following day. Antibodies and dilutions used were as follows: AMV-3C2 mouse monoclonal antibody raised against the ASLV viral matrix protein p19 (1:5; NICHD-funded Developmental Studies Hybridoma Bank maintained by The University of Iowa, Iowa City, IA); p27 rabbit polyclonal antibody raised against the ASLV viral capsid protein p27 (1:1000; Charles River Laboratories, Wilmington, MA). Antibody binding was routinely detected on the second day with Alexa 594 goat anti-mouse or Alexa 488 goat anti-rabbit antibodies (Molecular Probes, OR).

### Collection and processing of Amniotic Fluid samples

Amniotic fluid samples were collected at the time of embryo harvesting (ED) 6 to 8. Eggs were opened through the air chamber and amniotic fluid was collected with a 1 ml syringe and a 26 gauge × 3/8" needle. To ensure sterile conditions and avoid viral cross-contamination, eggs were wiped clean with alcohol wipes, forceps and scissors were flamed after each harvested embryo, and new sterile syringes and needles were used for each embryo. After collection, amniotic fluid samples from each embryo were divided in two and processed separately in order to obtain: i) cell-free amniotic fluid: amniotic fluid was centrifuged at 12,000 rpm for 15 min at 4°C, and the cell-free supernatant was used immediately or stored at -20°C until use; ii) cell-containing amniotic fluid: in order to keep cellular contents in the samples, amniotic fluid was not centrifuged and used immediately after harvest or stored at -20°C until use.

### Inoculation assay for detection of exogenous infectious viral particles in amniotic fluid

**Figure 1:**
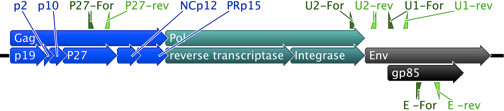
**ASLV genomic organization and target sites for primers used in this study**. This diagram represents the generic organization of ASLV viruses for the gag (blue) pol (green) and env (grey) genes and their respective subunits for proteins p19, p2, p10, p27, NCp12, PRp15, reverse transcriptase, integrase and gp85. Target sites for the primers are indicated by green triangles, dark green for forward and light green for reverse primers. The U1, U2 and P27 primer sets detect all viral subgroups, while the E primer set binds only to viruses of the E subgroup. Since differences exist between viral subgroups and even within members of the same subgroup this diagram is not drawn to scale.

DF-1 chicken fibroblasts cells were cultured and maintained according to ATCC instructions. On the day of seeding, DF-1 cells were inoculated with 100 μl of cell-free amniotic fluid from embryos from flocks 1-4 (n = 5 embryos/flock) or with cell-free amniotic fluid from embryos infected with RCAS-nucGFP virus as a positive control. Cells were passaged after 5 days in culture and maintained for an additional 5 days. Cells were then fixed in 4% paraformaldehyde in phosphate buffer for 20 minutes at room temperature and processed for IHC with the AMV-3C2 and p27 antibodies. In addition, presence of ASLV infective viral particles was tested by RT-PCR in samples of supernatant from inoculated DF-1 cells. DF-1 supernatant (1 ml) was collected 10 days after inoculation and centrifuged at 4°C and 12,000 rpm for 10 min to eliminate cellular content. Ten microliters of centrifuged DF-1 supernatant were treated with DNAseI (Invitrogen, Carlsbad, CA) and random primed reverse transcription was carried out with cloned AMV reverse transcriptase (Invitrogen, Carlsbad, CA); both reactions were performed according to the manufacturer instructions. Samples lacking AMV reverse transcriptase were run in parallel to control for possible "false positives" from DNA amplification. Two μl of RT-reaction product were used as template for PCR amplification with primers targeting the *hypervariable region *1 in the *gp*85 env gene, which is common to all ASLV subgroups including endogenous ALV-E and RCAS constructs (U1, Table 1 and Figure 1). PCR amplification was carried out with an initial cycle at 94°C for 2 min, followed by 40 cycles at 94°C for 30s, 60°C for 30s, and 72°C for 45 s and a final extension cycle at 72°C for 7 min.

**Table 1:** List of primers used in this study and target ASLV regions and subgroups

Primer	Target Region	Target ALV subgroup	Forward Primer 5'-3'	Reverse Primer 5'-3'	Product size (bp)	Reference
U1	gp85 *env*	A, B, C, D, J and E	CTRCARCTGYTAGGYTCCCAGT^1^	GYCAYCACTGTCGCCTRTCCG^1^	229	[14]
U2	*pol-env*	A, B, C, D, J and E	GGATGAGGTGACTAAGAAAG	GGGAGGTGGCTGACTGTGT	295-326^2^	[24]
E	gp85 E *env*	E	GGCTTCGCCCCACACTCCAA	GCACATCTCCACAGGTGTAAAT	265	[14]
P27	p27 protein	A, B, C, D, J and E	CAGGCCGCATTATTAAGACC	TGGCTGTGACTTCTGCTGCCT	240	This study

^1^R = A/G; Y = T/C

^2^Exact size depends on the ALV subgroup.

### RT-PCR assay for detection of endogenous infectious viral particles in amniotic fluid

Cell-free amniotic fluid from embryos from flocks 1-4 (n = 5 embryos/flock) was assayed by RT-PCR for the detection of endogenous viral particles. Embryos infected with RCAS-nucGFP virus were used as positive controls (n = 5). Ten microliters of centrifuged amniotic fluid were treated with DNAseI (Invitrogen, Carlsbad, CA) and random primed reverse transcription was carried out with cloned AMV reverse transcriptase (Invitrogen, Carlsbad, CA); both reactions were performed according to the manufacturer instructions. Samples lacking AMV reverse transcriptase were run in parallel to control for possible "false positives" from DNA amplification. Two μl of reverse transcribed reaction (or reactions lacking reverse transcriptase) were used as template for PCR reactions designed to amplify regions common to all ASLV subgroups, including endogenous ALV-E and RCAS constructs (primer sets U1 and U2, Table 1 and Figure 1). Amplification with the U1 primer set was done as described above. For the U2 primer set a 'touch down" PCR program with the following parameters was used: 14 rounds of PCR using 1 min denaturation at 93°C, 1 min annealing beginning at 60°C and decreasing 1°C in each cycle, and 1 min 30s extension at 72°C; followed by 30 cycles of 93°C for 1 min, 48°C for 1 min, 72°C for 1 min 30s.

### Polymerase Chain Reaction screening for ASLV ev loci

Ten μl of cell-containing amniotic fluid samples from embryos from flocks 1-4 (n = 5-8 embryos/flock) were used as template for PCR screening of *ev loci *composition. Three sets of primers were used to target different regions of the *ev loci*: i) the region corresponding to the p27 protein; ii) a 200 bp region of the pol-env junction; and iii) a region of the gp85 glycoprotein specific for subgroup E (P27, U2 and E respectively, Table 1 and Figure 1). For the P27 and E primer sets PCR amplification was carried out with an initial cycle at 94°C for 2 min, followed by 40 cycles at 94°C for 30s, 60°C for 30s, and 72°C for 45 s and a final extension cycle at 72°C for 7 min. For U2 primers the 'touch down' PCR program described in the previous section was used.

## Results

### SPF chicken embryos expressed ASLV-related viral proteins

**Figure 2:**
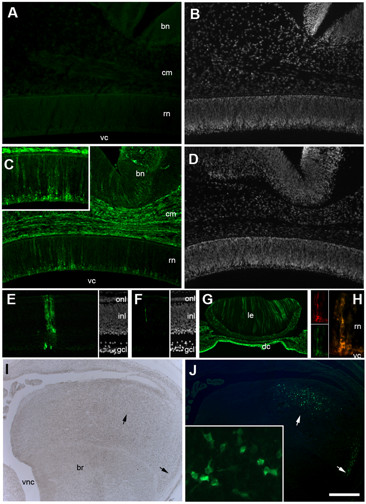
**Immunohistochemical detection of ALV viral proteins in certified SPF chicken embryos**. (A-D) Transversal sections of ED6 chick embryo heads, processed by IHC against p27 viral protein (A and C) and DAPI (B and D). (A-B) Section corresponding to an embryo from Flock 1 that was negative for endogenous viral protein expression. (C-D) Section corresponding to a similar region of another embryo from Flock 1, showing extensive expression of p27 viral protein in the retinal neuroepithelium (rn), surrounding cephalic mesenchyme (cm) and brain neuroepithelium (bn) (vc: vitreal cavity). (E and F) Transversal sections of the retina of two different ED11 embryos from Flock 4 showing discrete columns of p27 positive cells; notice in F that in some cases only a few cells are positive for endogenous viral proteins (onl: outer nuclear layer; inl: inner nuclear layer; gcl: ganglion cell layer). (G) Transversal section through the lens of an ED6 embryo from Flock 2 showing endogenous expression of p27 viral protein (le: lens; dc: developing cornea). (H) Transversal section of the retina of an ED6 embryo from Flock 1 showing co-expression of p19 (red) and p27 (green) (rn: retinal neuroepithelium; vc: vitreal cavity). Notice the columnar pattern of expression, particularly in the retinal neuroepithelium and the lens. (I-J) Transversal section through the brain of an ED11 embryo from Flock 4; (I) bright field image; (J) immunostaining for p27 viral protein. Small, well defined areas of the developing brain (br) showed positive expression of p27 (arrows); these areas showed a symmetrical pattern, being present in both, right and left brain structures (vc: ventricular cavity). Scale bar in (J) corresponds to 150 μm for A-D; 100 μm for E-F and H; 250 μm for G and 450 μm for I-J.

Immunohistochemical analysis of head sections revealed endogenous expression of both, p19 and p27 viral proteins in embryos from 3 of the 4 flocks tested (Table 2). The percentage of embryos that tested positive for p19 and p27 varied among these flocks, ranging from 33% to 100% (Table 2). In addition, the extent of viral protein expression also varied between the different flocks. Embryos from flocks 1 and 2 that tested positive for p19 and p27 showed extensive expression in the retina, brain and cephalic mesenchyme (Figure 2A-D) while embryos from flock 4 showed a much more restricted expression pattern, confined to discrete columns in the retina, sometimes with as few as 2 to 3 cells per column (Figure 2E and 2F) and small regionally restricted areas of the brain (Figure 2I and 2J). It is worth noting that processing of several serial sections per embryo was critical in order to identify these very discrete areas of endogenous viral protein expression in embryos from flock 4. In addition, embryos from flocks 1 and 2, but not 3 and 4, showed significant expression of viral proteins in the lens (Figure 2G). In all the flocks that tested positive for viral proteins, the pattern of expression in the retina and lens resembled the columnar pattern characteristic of retroviral infection in these tissues (Figure 2). Double labeling IHC confirmed co-expression of p19 and p27 protein in all cases (Figure 2H).

**Table 2:** Immunohistochemical detection of endogenous viral proteins in SPF embryos

Flock	Total n	p27/p19 (+)	% of (+) embryos
**1**	8	4	50
**2**	4	4	100
**3**	6	0	0
**4**	9	3	33

Serial sections (1 every 5/embryo) were processed by immunohistochemical detection of endogenous p27 and p19 viral proteins. Three of the four flocks tested showed embryos positive for endogenous viral expression. The percentage of positive embryos varied among the flocks. In all positive embryos, p27 and p19 were co-expressed.

### SPF embryos expressing ASLV viral proteins were negative for exogenous and endogenous ALV infective viral particles

**Figure 3:**
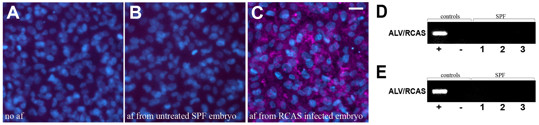
**Detection of infective ASLV viral particles in amniotic fluid from SPF chicken embryos**. (*A-D) Inoculation assay for detection of exogenous ASLV infection*. DF-1 chicken fibroblast cells cultured in the presence of (A) no amniotic fluid; (B) amniotic fluid from untreated SPF chicken embryo; and (C) amniotic fluid from SPF chicken embryo infected with RCAS-nucGFP construct. Inoculated cells were processed for immunohistochemical detection of ASLV viral protein p27 (red) and counterstained with DAPI (blue). Lack of immunohistochemical signal in cells inoculated with amniotic fluid from SPF untreated embryos (B) suggested that SPF embryos were not pre-infected with exogenous ASLV virus. (A) and (C) were used as negative and positive controls respectively. af: amniotic fluid. Scale bar:20 μm. (D). RT-PCR analysis of supernatants from (-) non-inoculated DF-1 cells; or DF-1 cells inoculated with (+) amniotic fluid from SPF embryos infected with RCAS-nucGPF; or (1-3) untreated SPF embryos, confirmed absence of exogenous viral infection in SPF embryos. *(E) Detection of endogenous infective viral particles directly from amniotic fluid*. Amniotic fluid from (+) SPF embryos infected with RCAS-nucGPF; or (1) untreated SPF embryos negative for p19/p27 by IHC; or (2-3) untreated SPF embryos positive for p19/p27 by IHC, was used for direct RT-PCR detection of infective ASLV viral particles. Positive RT-PCR amplification from amniotic fluid from embryos purposely infected with RCAS-nucGFP (+) set proof-of-principle for the approach. On the other hand, lack of RT-PCR amplification in amniotic fluid from SPF untreated embryos further demonstrated absence of both, exogenous and endogenous ASLV infective viral particles. (-) RT-PCR negative control.

These observations suggested that embryos from at least three of the flocks under study may have been either i) pre-infected by exogenous ASLV or ii) have an *ev loci *capable of producing infectious endogenous viral particles. In order to rule out the first possibility, we designed a series of experiments directed at identifying live exogenous viral particles in the amniotic fluid of the chick embryos. For this purpose, we took advantage of the DF-1 cell line, a chicken fibroblast cell line derived from the chicken endogenous *ev loci-free *East Lansing Line 0 (also known as ELL-0 or EV-0 line). Since these cells do not express the subgroup E membrane receptor TVB*S1, they are resistant to ALVE but susceptible to infection from all exogenous ASLV subgroups [9,11-13]. DF-1 cells were inoculated with 100 μl of amniotic fluid from embryos from flocks 1-4 (n = 5/flock) or with amniotic fluid from positive control embryos infected with RCAS-nucGFP virus. Similar to untreated control DF-1 cells (Figure 3A), cells inoculated with amniotic fluid from embryos from flocks 1-4 were negative for p19 or p27 viral proteins, with the exception of those inoculated with amniotic fluid from RCAS-nucGFP infected embryos (Figure 3B-C). In order to rule-out the possibility of viral infection below the level of immunohistochemical detection, we also carried out the more sensitive RT-PCR assay for detection of ASLV viral particles in the supernatant of amniotic fluid-inoculated DF-1 cells. In agreement with the immunohistochemical results, RT-PCR with a pan-ASLV primer set (recognizing all exogenous ASLV subgroups and RCAS virus, Table 1 and Figure 1) was negative in all cases with the exception of positive controls (Figure 3D). Taken together, these results provide compelling evidence that embryos from flock 1-4 were not pre-infected with exogenous ASLV viruses.

The next step was to test whether SPF chick embryos were producing viral particles from endogenous *ev loci*. Since the previous set of experiments clearly showed that SPF embryos were devoid of infection by exogenous ASLVs, detection of ASLV viral RNA in fluid-components of the eggs would indicate production of endogenous viral particles. Current techniques involve RNA purification from egg albumin samples for RT-PCR screening [14]. This approach, however, requires cumbersome procedures due to coagulation of albumin proteins and may also lead to false negatives as a consequence of possible low yield RNA recovery during purification [14]. In order to overcome these limitations, we developed an assay to test for viral particles directly from amniotic fluid samples. We reasoned that the amniotic fluid of embryos infected with viruses should contain viral particles, and that since amniotic fluid is 98-99% water [15] it should be compatible with RT-PCR reactions without the need for RNA isolation. In order to eliminate cells and DNA content from the amniotic fluid, samples were centrifuged and treated with DNAse as described in Methods. Cell-free amniotic fluid was used as template for RT-PCR detection of viral RNA with primers recognizing all ASLV subgroups, including the endogenous ALVE subgroup and the related RCAS virus (Table 1 and Figure 1). Successful amplification from amniotic fluid of embryos infected with RCAS-nucGFP, even in the case of embryos injected only 24 hours before amniotic fluid harvest, demonstrated the efficiency and sensitivity of the new assay (Figure 3, E). In contrast, amplification products from amniotic fluid samples from flocks 1-4 were consistently negative, including embryos that were positive for immunohistochemical detection of p19/p27 proteins (Figure 3E). Thus, although viral proteins could be detected in tissues of a significant percentage of embryos from flocks 1-4, there was no evidence of viral particle production. These results are consistent with our observations from the inoculation assays, and further confirmed that embryos from flocks 1-4 were not pre-infected with exogenous ALV viruses. In addition, these results also demonstrated that the embryos were not producing viral particles from endogenous *ev loci*.

### Genetic screening demonstrated that SPF embryos have ev loci encoding the p27 viral protein

The observation that SPF embryos were not infected by either exogenous or endogenous ASLV virus, contrasts with our findings showing detection of ASLV viral protein in vivo by IHC. This apparent contradiction likely suggests that expression of the viral proteins in tissues may, in some cases, be independent of the production of viral particles. We therefore reasoned that although the *ev loci *present in embryos from the flocks analyzed might not be capable of generating viral particles, they may still be capable of producing viral proteins such as p27 and p19. This premise prompted further molecular analysis of their genomic composition. Since amniotic fluid normally contains embryonic cells, we decided to use amniotic fluid samples with no centrifugation or DNAse treatment to screen for the presence of three different regions of the *ev loci *in the genome of embryos from flocks 1-4 by PCR amplification. Primers were designed to target: i) the region corresponding to the p27 protein; ii) a 200 bp region of the pol-env junction; and iii) a region specific for the subgroup E viral envelope (P27, U2 and E respectively, Table 1 and Figure 1). Results are summarized in Table 3. All three *ev loci *regions were observed in all flocks analyzed and they showed a similar pattern across flocks, with a subpopulation of embryos with *ev loci *containing all three regions, and other subpopulations with variable combinations of two or only one of the regions included in this screening. Strikingly, almost all the embryos tested (25/26) were positive for the p27 *ev loci *region. Most importantly, these results demonstrated a high level of heterogeneity of the *ev loci *genotype among embryos of the same flock. As also shown in Table 3, we could not determine any obvious correlation between presence or absence of the *ev loci *regions analyzed by RT-PCR and detection of p27 expression by IHC.

**Table 3:** Detection of genomic *ev loci *regions in SPF embryos by PCR

Flock	Embryo	p27 region	Pol-env junction	Gp85 E env	IHC
**1**	1	+	+	+	na
	2	+	-	-	na
	3	+	+	-	na
	4	-	-	-	na
	5	+	+	-	na

**2**	1	+	+	+	+
	2	+	-	-	+
	3	+	+	-	na
	4	+	+	-	na
	5	+	+	+	na
	6	+	+	-	na

**3**	1	+	+	-	-
	2	+	+	+	-
	3	+	+	+	-
	4	+	+	+	-
	5	+	+	+	-
	6	+	+	+	na
	7	+	-	+	na
	8	+	+	-	na

**4**	1	+	+	-	-
	2	+	-	-	+
	3	+	+	+	-
	4	+	+	+	+
	5	+	+	+	-
	6	+	+	-	+
	7	+	+	+	-

Amniotic fluid samples were used to screen for the presence of three different regions of the *ev loci *by PCR. Presence of these sequences in the genome varied between flocks and between embryos among the same flock. Some embryos showed all three *ev loci *regions while others showed different combinations of two or only one of the regions. Twenty-five out of twenty-six embryos were positive for the p27 DNA region. No obvious correlation between composition of *ev loci *and detection of p27 expression by immunohistochemistry could be determined. IHC: immunohistochemistry. na: not available.

## Discussion

The results of this study can be summarized as follows: i) expression of endogenous ASLV viral proteins p19 and p27 in tissues was detected in certified SPF chicken embryos; ii) the extent of viral protein expression as well as the percentage of positive embryos varied among the different flocks analyzed; iii) the pattern of viral protein expression in retina and lens resembled the columnar pattern characteristic of retroviral infection in these tissues; iv) SPF embryos expressing ASLV viral proteins in tissues were negative for exogenous and endogenous ASLV infective viral particles; v) genetic screening demonstrated that SPF embryos from all flocks analyzed contained several *ev loci *regions; vi) *ev loci *composition varied significantly between flocks and between embryos of the same flock; and vii) almost all the embryos tested (25/26) were positive for the p27 *ev loci *region.

SPF commercial chicken lines are certified free of ASLV virus by screening for expression of the p27 protein using a standard enzyme-linked immunosorbent assay (ELISA) [8,16]. This technique allows detection of infectious viral particles present in biological fluids such as meconia, swab exudates, blood, serum and albumin from ASLV infected chickens [16]. However, commercial breeders use ELISA tests according to vaccine production standards [17], which do not necessarily ensure eggs suitable for research using RCAS retroviruses. In most commercial breeders, all birds are bled when a colony is established, and thereafter only 5% of the birds are bled each month; blood samples are pooled together and the resulting serum samples are screened for antibodies to the relevant pathogens [17]. Therefore, while this approach may be useful for the detection of high viral titers associated with widespread exogenous ASLV infection, it may not be sensitive enough as to detect a few positive embryos among the tested pool. Additionally, the genome of nearly all chickens contains various DNA proviral insertions belonging to the subgroup E ASLV. Only a few chicken lines are free of *ev loci *[9], and unfortunately, most producers of SPF eggs do not use these lines because their egg production, fertility and embryo survival are significantly inferior [7,18].

Our study demonstrates that most commercial SPF chicken strains contain transcriptionally active *ev loci*. Three out of the four SPF chicken flocks tested in this study were positive for ASLV viral proteins p19 and p27, even though they were negative for infectious viral particles by ELISA (as certified by the breeders) and our own analyses by RT-PCR in amniotic fluid samples and supernatant from inoculation assays. The importance of these observations for the developmental research community is highlighted by the fact that, based on a survey of the literature over the last 10 years, about 60% of the articles in which SPF chick embryos and RCAS vectors were used, based their analyses and conclusions upon identification of infected cells/tissues by IHC for viral proteins p19 and p27. These studies were done under the assumption that, since SPF chick embryos are certified to be free of ASLV viral infection, they should not express viral proteins in tissues. Contrary to this, *our results demonstrate that lack of ASLV viral infectious particles does not necessarily correlate with lack of viral protein expression in SPF chick embryo tissues*.

The ability of some *ev loci *to express viral proteins to detectable levels by IHC in the absence of infectious viral particles, makes it imperative that researchers understand the biology of these events and its consequences for the research field. Some aspects of the biology of ALVE retroviruses that are particularly relevant in this context are as follows:

i) *ALVE loci *in White Leghorn Chickens include at least 23 known *loci *consisting of both defective and non-defective retroviral inserts, ranging from a single LTR region such us *ALVE15*, to full length retrovirus genomes such as *ALVE1, 2, 7, 10, 11, 12, 14 *and *21 *([8] and references therein).

ii) Expression of a given *ev locus*, as well as its level of expression, depends on several factors such as the completeness of the proviral genome, its site of integration and epigenetic modifications. The *ALV1 locus *for example, although present in most White Leghorn Chicken lines is normally silent; however under certain conditions, such as treatment with methylation inhibitors, *ALV1 *is capable of producing viral particles [19].

iii) The genetic background in which a given *ev locus *is found is also important in determining its expression. SPF chicken lines can be fixed (every individual in the population has the same allele at a particular locus), segregating (different alleles are present in different individuals of the population), or both, for several different *ev loci *as well as for the TVB* loci, which encode the different ASLVE membrane receptors TVB*S1, TVB*S3 and TVB*R [8]. Breeding of chickens with different genetic background may give rise to higher levels of *ev loci *expression, and even de-novo production of infectious viral particles. The chicken line 0.44-VB*S1-EV21, for example, is fixed for the *ALVE 21 locus *but segregating for the TVB*S1 and TVB*S3 receptor alleles. *ALVE21 *in a TVB*S3 background is essentially silent, while in a TVB*S1 background is capable of producing high levels of infectious viral particles [8].

The SPF breeder lines are often crossed to gain the F1 vigor needed to generate the number of fertile eggs required by regulatory agencies [8]. The crossing of lines fixed or segregating for different *ev *and/or TVB* loci, increases the chances for stochastic genomic recombination, thus consequently increasing genomic heterogeneity for *ev loci *in the chick population. Furthermore, even within a given established chicken line, heterogeneity for *ev loci *composition can give rise to variations in the genetic background, and in turn in *ev loci *expression, from flock to flock and even within a same flock. In addition, chicken flocks have limited productivity (30-40 weeks, [20]; B&E Eggs personal communication), which means that even when receiving embryos of a given line and breeder, periodic variations in the flock source are inevitable.

**Figure 4:**
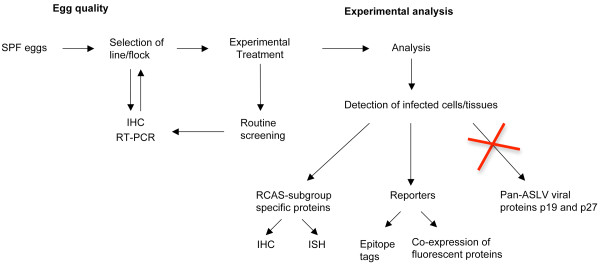
**Recommended workflow for developmental studies involving chicken embryos and RCAS vectors**. SPF eggs should be selected by screening for lack of ASLV viral particles and endogenous viral protein expression in tissues. Afterwards, routine screening of selected lines is necessary. During experimental analysis, experimental infection should be determined by expression of viral proteins specific for the subgroup corresponding to the RCAS vector used, or alternatively, by using expression reporters such as epitope tags or fluorescent proteins.

Our observations demonstrating high levels of heterogeneity for viral protein expression in tissues as well as for *ev loci *composition, even in embryos belonging to the same flock, clearly point out the need for raising awareness among the developmental biology community. Retrospectively, our results suggest that studies in which infected cells and tissues were identified simply by viral protein expression without controlling for possible endogenous expression of those proteins may need to be considered with caution. As for future studies, we recommend a series of practical guidelines to ensure quality of research animals and accuracy of the interpretation of results. The rationale and usefulness of these guidelines are discussed below and a summarized schematic representation of them is presented in Figure 4.

### Practical guidelines for developmental studies involving chicken embryos and RCAS vector

A continuous and dynamic process involving both egg quality testing and the use of appropriate procedures during experimental analysis is necessary.

#### Egg quality

*i) careful characterization and selection of chicken strains for experimental use*. A source (line/flock) of research quality SPF eggs should be selected by screening for lack, or minimal detection if otherwise not possible, of ASLV viral particles and endogenous viral protein expression in tissues by, for example, RT-PCR and IHC respectively. Here we propose a straightforward RT-PCR assay for detection of infectious viral particles directly from chick amniotic fluid that can be completed in only a few hours. Additionally, we strongly recommend that chicken lines be also tested for expression of viral proteins by IHC. Based on our observations, particularly from flocks 1 and 4, this screening should be as thorough as possible, since analysis of a few embryos per flock as well as analysis of a few sections per embryo may in some cases give rise to "false negatives". This type of screening seems of special relevance for experimental settings in which the effects of a particular treatment is assessed in dissociated or cultured cells. In this situation, the lack of surrounding tissues, that make possible comparisons between RCAS infected and contralateral non-infected structures, makes identification of endogenous viral protein expression almost impossible.

*ii) Routine screening of SPF lines/flocks in use*: Routine screening by RT-PCR and IHC is necessary to ensure that selected lines and flocks in use do not become ASLV positive. If at any given point, de-novo ASLV viral particle production and/or viral protein expression in tissues is detected, a new line or flock should be tested and selected for further use.

#### Experimental Analysis

*Use of alternative methods, other than immunohistochemical detection of pan-ASLV viral proteins such as p19 and p27, for identification of experimentally infected cells and/or tissues*. Considering that the standard tests used by SPF egg breeders are reliable in ensuring eggs free of exogenous ASLV viruses, and that the most commonly used RCAS constructs belong to the exogenous subgroups A-D, techniques such as IHC and in situ hybridization to detect ASLV subgroup specific proteins would be ideal. Hunter and collaborators reported the generation of a mouse monoclonal antibody directed against the RSV subgroup A Env protein [21], but to our knowledge, the only subgroup specific antibodies commercially available are the monospecific polyclonal chicken antibodies against the RSV subgroups A and B from Charles River. Although we were initially concerned about possible non-specific staining of these chick antisera in chick tissues, the Avian Leukosis RSV A antisera (catalog number: 523501) has worked very well in our hands and we now use it routinely to detect infected cells in experimental animals (unpublished results; antibody concentration 1:5000). Further development of antibodies covering the whole spectrum of ASLV subgroups would be highly beneficial for the research community. Alternatively, the nucleotide sequences of the gp85 envelope region, which defines the ASLV subgroups, have been well characterized and can be used for designing subgroup specific probes for *in situ *hybridization when appropriate antibodies are not available ([22] and references therein).

Another alternative is to express the gene of interest fused to an epitope tag, or to co-express it with a fluorescent reporter. RCAS derivatives that allow the insertion of genes using the Gateway recombination cloning system have been generated [23] and are available to the research community from AddGene http://www.addgene.org. Some of these constructs have been developed to allow expression of the gene of interest with an influenza hemaglutinin (HA) epitope tag, either in the amino (NHA) or carboxy (COOH) terminus. Alternatively, co-expression of fluorescent reporters together with the gene of interest could be considered for small size genes, but due to inherent limitations to the carrying capacity of the RCAS vector backbone (insert sizes bigger than 2.5K are unstable, [3]) they cannot be used for long gene coding sequences.

## Conclusions

We have demonstrated that certified SPF chicken embryos have transcriptionally active endogenous *ASLV loci* capable of expressing ASLV viral proteins even when those *loci *are not capable of producing infectious viral particles. We also show that the extent of viral protein expression in embryonic tissues varies not only among flocks but also between embryos of the same flock. In addition, our genetic screening revealed significant heterogeneity in *ev loci *composition even among embryos of the same flock. These observations point to the need for systematic screening of chicken lines and flocks as well as appropriate methodology to identify experimentally infected cells and tissues in developmental studies using the chick as the experimental model.

## Competing interests

The authors declare that they have no competing interests.

## Authors' contributions

MM, KJW and MVCS designed and performed experiments and analyzed the data. MVCS wrote the paper and MM and KJW contributed with critical reading and editing of the text. All authors read and approved the final manuscript.
